# Contextuality and Informational Redundancy

**DOI:** 10.3390/e25010006

**Published:** 2022-12-21

**Authors:** Ehtibar N. Dzhafarov, Janne V. Kujala

**Affiliations:** 1 Department of Psychological Sciences, Purdue University, West Lafayette, IN 47907, USA; 2 Department of Mathematics and Statistics, University of Turku, FI-20014 Turun yliopisto, Finland

**Keywords:** contextuality, consistent connectedness, connections, functions of connections, measurements, non-measurements

## Abstract

A noncontextual system of random variables may become contextual if one adds to it a set of new variables, even if each of them is obtained by the same context-wise function of the old variables. This fact follows from the definition of contextuality, and its demonstration is trivial for inconsistently connected systems (i.e., systems with disturbance). However, it also holds for consistently connected (and even strongly consistently connected) systems, provided one acknowledges that if a given property was not measured in a given context, this information can be used in defining functions among the random variables. Moreover, every inconsistently connected system can be presented as a (strongly) consistently connected system with essentially the same contextuality characteristics.

## 1. Introduction

Throughout this note, we use the standard language and notation of the Contextuality-by-Default theory (CbD) [1]. Familiarity with CbD therefore is desirable, although we will provide the meaning of all the terms and notions we use. As a throughout example to illustrate the issues, consider a rank-3 cyclic system of random variables [2],
(1)
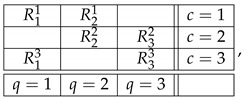

where $R_{q}^{c}$ is the random variable representing the outcome of measuring content *q* (that which is being measured) in context *c* (conditions under which it is measured). The random variables within each context (e.g., $R_{1}^{1},R_{2}^{1}$) form a *jointly distributed bunch*, while any two random variables in two different contexts (e.g., $R_{1}^{1},R_{1}^{3}$ or $R_{1}^{1},R_{3}^{2}$) are *stochastically unrelated* (possess no joint distribution). The system is *consistently connected* (has no disturbance) if the distribution of the variables sharing a content is always the same. In CbD, systems are generally *inconsistently connected*.

The immediate motivation for this paper is the appearance of an abstract [3] claiming that the extension of the notion of contextuality to inconsistently connected systems, such as offered by CbD, is impossible, because it contradicts the conjunction of certain “core principles”. Some of these principles are satisfied in CbD trivially. One of them, however, related to creating new random variables as functions of the old ones, is known not to hold. The reasons for this are well-understood and presented in the CbD literature (e.g., in Ref. [1]). It is not surprising therefore that if one posits this “principle” as a principle, it rules out CbD. Moreover, we show in this paper that, with careful formulations of the concepts involved, the “principle” in question also contradicts more traditional approaches to contextuality, those confined to consistently connected systems. In other words, the alleged principle is in conflict with the very notion of contextuality, rather than just CbD or other extensions of contextuality to inconsistently connected systems.

The purpose of this paper, however, is not entirely polemical. Our analysis leads us to a better understanding of the structure of the systems of random variables, specifically of what information in them can be accessed and utilized in defining new random variables based on the old ones.

## 2. Connections and Their Functions

A *content-context matrix*, like (1), typically contains empty cells, or “non-measurements,” representing cases when a given content is not measured in a given context. In CbD, the empty cells can always be viewed as containing deterministic variables [4] (those equal to some value with probability 1). For instance, (1) can be presented as
(2)
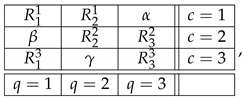

where α stands for a random variable $R_{3}^{1}$ such that $p\left[R_{3}^{1}=\alpha \right]=1$ (and analogously for β,γ). This system is equivalent to (1) in the sense that their contextuality status and the degree of contextuality in them are precisely the same. In more traditional approaches to contextuality, confined to consistently connected systems, filling of the empty cells with deterministic variables is not allowed. Nevertheless, the empty cells in any system, even if consistently connected, provide certain information (“no measurement output in this cell”), and this information can be utilized when one introduces new variables as functions of old ones. This simple observation is central for the present paper.

In CbD, the random variables sharing a content are referred to as *connections* (between bunches). In Ref. [3], connections are called *observables*. The latter term is standard in quantum mechanics, but its use is not natural in CbD, for four reasons. *First*, CbD has multidisciplinary applications, and outside quantum mechanics the term “observable” may not be well-defined. *Second*, and most important, observables are defined by contents only (by what is being measured), while in CbD contexts play an equally important role—which is why the distributions within a connection are generally different in different contexts. Even in quantum mechanics, the random variables in a connection are generally determined not only by a quantum observable but also by factors, such as quantum states and signaling, that depend on contexts. See, for example, the analysis of “signaling in time” in Refs. [5,6] in sequential quantum measurements (where later measurements can be affected by settings for the previous ones). *Third*, unlike the bunches of random variables within contexts, connections are not random vectors, as they consist of pairwise stochastically unrelated variables. *Finally*, as explained in Section 9, connections in CbD can be redefined in multiple ways without affecting the contextuality status of a system (i.e., preserving the degree of contextuality in it, by conventional measures). As a result, one can have contextually equivalent systems with very different sets of connections.

We will adopt the notation $R_{q}$ for the connection corresponding to content *q* (e.g., $R_{1}$ for q=1, $R_{2}$ for q=2, etc.). Note that the use of the script letter R is to indicate that a connection possesses no joint distribution of its elements. In view of the simple observation above, we will expand the definition of a connection here to also include non-measurements. Thus, in our example,

(3) $$R_{1}=\begin{array}{c} R_{1}^{1}\\ □\\ R_{1}^{3} \end{array},R_{2}=\begin{array}{c} R_{2}^{1}\\ R_{2}^{2}\\ □ \end{array},R_{3}=\begin{array}{c} □\\ R_{3}^{2}\\ R_{3}^{3} \end{array}\;,$$

where □ stands for the dummy variable whose single value is “no measurement output.” We can now define the notion of some connection being a function of some *k* connections. Without loss of generality, let them be $R_{0}$ and $\left(R_{1},\ldots R_{k}\right)$, respectively. We will say that

(4) $$R_{0}=f\left(R_{1},\ldots R_{k}\right),$$

if in every context *c*,

(5) $$R_{0}^{c}=f\left(R_{1}^{c},\ldots ,R_{k}^{c}\right),$$

with the understanding that if a variable $R_{q}^{c}$ is undefined, it is replaced with □. In CbD, one can simply view □ as the single value of a deterministic variable, $R_{q}^{c}=□$, but in view of the approaches confined to consistently connected systems, it is better to treat the random variables in a system and its □ entries separately. For a given context *c*, let us agree to write $R_{q}=a$ if $R_{q}^{c}=a$ or if a=□ in the $\left(q,c\right)$-cell.

(We need to add a technical detail here. In CbD, all variables within a connection should have the same set of possible values. Therefore, if in $R_{1}$ the variables $R_{1}^{1}$ and $R_{1}^{3}$ are dichotomic, ±1, then they have to be redefined as having values $\left\{1,-1,□\right\}$, with the understanding that $p\left[R_{1}^{1}=□\right]=p\left[R_{1}^{3}=□\right]=0$. The non-measurement variable $R_{1}^{2}$ should also be redefined as having values $\left\{1,-1,□\right\}$, with $p\left[R_{1}^{2}\neq □\right]=0$.)

## 3. From Noncontextual to Contextual Systems

Let X,Y,Z be ±1-variables with $p\left[X=1\right]=p\left[Y=1\right]=p\left[Z=1\right]=1/2$, and let them be pairwise stochastically unrelated. The variant of the system (1) shown below is consistently connected:(6)
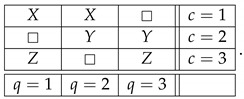
This system is noncontextual because it has a *coupling* with *(multi)maximal connections*:(7)
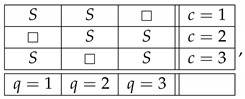

where *S* is a ±1-variable with $p\left[S=1\right]=1/2$. By definition, it is a coupling of (6) because (a) all variables in it are jointly distributed, and (b) in every context the joint distribution of the variables is the same as in system (6). This coupling has (multi)maximal connections because in each connection all the random variables are pairwise equal to each other with maximal possible probability (which in this example, as in all consistently connected systems, is 1). (The prefix “multi” is to indicate that the maximal probability is achieved for all pairs of the random variables in a connection, but since in our example a connection contains only two variables, the notions of multimaximality and maximality coincide.)

Let us add a new connection $R_{4}$ to this system:(8)
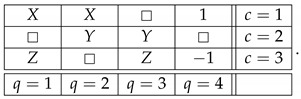
The system remains noncontextual, because, as mentioned earlier with reference to [4], the contextuality status of a system does not change if one adds to or deletes from it deterministic variables. The new system, however, is no longer consistently connected (because being equal to 1 with probability 1 and being equal to −1 with probability 1 describes two different distributions). Let us further introduce another connection, $R_{0}$:(9)
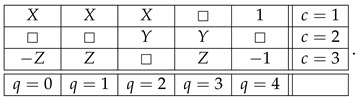
Observe now that $R_{0}$ can be obtained as a function of other connections in the system,

(10) $$R_{0}=\left\{\begin{array}{ccc} R_{1} & \mathrm{if} & R_{4}=1\\ -R_{1} & \mathrm{if} & R_{4}=-1\\ □ & \mathrm{if} & \mathrm{otherwise} \end{array}\right\}=f\left(R_{1},R_{4}\right).$$

Note that “if” here means “in any context c in which”. As a function of other connections, therefore, $R_{0}$ is *informationally redundant*, and the authors of Ref. [3] think this means that the addition of $R_{0}$ to the noncontextual system (8) should leave the system noncontextual. However, the new system is contextual. This is easy to see by considering its subsystem
(11)
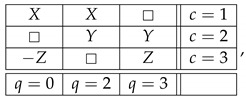

which is a PR box of rank 3, a system whose degree of contextuality is maximal among all cyclic rank-3 systems in any reasonable theory of contextuality.

## 4. Why Is This Happening in CbD?

What shall one do now with the expectation that (9) should not have been contextual because $R_{0}$ is informationally redundant? Its plausibility depends on the definition of contextuality, because this expectation clearly does not hold for just any property of the system. Think, e.g., of such properties as the number of connections in the system, or the product of the random variables within a given context. To give a more remote but apt analogy, consider the notion of a rank of a matrix. Adding to a matrix a new column whose entries are squared values of one of the old columns would generally increment its rank by 1, even though the new column is informationally redundant.

It just happens that contextuality is one of such properties. Contextuality is not about predicting values of random variables in a system. Rather it is about the compatibility of certain couplings for its connections with the distributions of its bunches. The answer as to why such compatibility generally changes as one adds new variables to the system, even if computable from other variables, has been given in Ref. [1], and we will present it using our example. The system in (8) is noncontextual because it has a coupling with (multi)maximal connections, In fact, this coupling happens to be unique:(12)
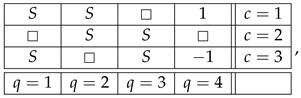

where *S* is as in (7). It is easy to see that the only coupling of $R_{0}$ compatible with coupling (7) is
(13)
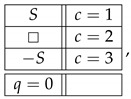

and this is clearly not a (multi)maximal coupling: $p\left[S=-S\right]=0$, whereas in the maximal coupling of $R_{0}$ (since *X* and −Z are identically distributed) the probability would be 1. This means that there exists no coupling of the entire system (9) in which all connections are (multi)maximal, and this, by definition, means that the system is contextual.

## 5. What If All Systems Are Consistently Connected?

The question one can pose now is: how critical is it that in order to transform the noncontextual system (6) into the contextual system (9) we have created a system, (8), which is inconsistently connected? The answer is that it is not critical at all. To see this, let us modify our example, and replace (8) with
(14)
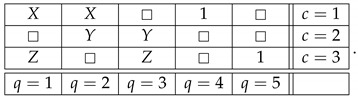
This system is noncontextual and consistently connected, but we can still introduce the same connection $R_{0}$ as before by means of the following function of the existing connections:

(15) $$R_{0}=\left\{\begin{array}{ccc} R_{1} & \mathrm{if} & R_{4}=1\\ -R_{1} & \mathrm{if} & R_{5}=1\\ □ & \mathrm{if} & \mathrm{otherwise} \end{array}\right\}=g\left(R_{1},R_{4},R_{5}\right).$$

We end up with essentially the same contextual system as (9), but one satisfying the condition of consistent connectedness:(16)
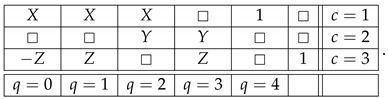


## 6. Strong Consistent Connectedness

In the cause of defending the “core principle” of informational redundancy, one could point out that the last system, while consistently connected, is not *strongly* consistently connected [7], because it contains the subsystem
(17)
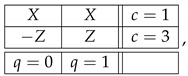

in which the bunches $\left(X,X\right)$ and $\left(-Z,Z\right)$ are not identically distributed. In a strongly consistently connected system, if two contexts contain variables with the matching contents, say,

(18) $$\begin{array}{c} R_{1}^{c},\ldots ,R_{k}^{c}\\ R_{1}^{c^{\prime }},\ldots ,R_{k}^{c^{\prime }} \end{array},$$

their joint distributions are the same. This constraint, for instance, is imposed in the sheaf-theoretic approach to contextuality [8].

It is a debatable point whether consistent connectedness, which is not strong, should also be considered a form of disturbance, but we can avoid this discussion. Examples with all systems involved being strongly consistently connected can be readily constructed. Consider the following noncontextual system,
(19)
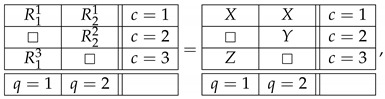

where X,Y,Z have the same properties as before. Define a new connection $R_{0}$ as follows:

(20) $$R_{0}=\left\{\begin{array}{ccc} R_{2} & \mathrm{if} & R_{1}=□\\ -R_{1} & \mathrm{if} & R_{2}=□\\ □ & \mathrm{if} & \mathrm{otherwise} \end{array}\right\}=\phi \left(R_{1},R_{2}\right).$$

The new system
(21)
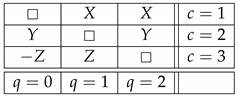

is contextual. In fact, it is again a PR box of rank 3, like the one we had in (11). Both the initial system (19) and the resulting system (21) are strongly consistently connected.

## 7. Informationally Interchangeable Connections

Returning to the example of Section 5, it also can be modified to involve only strongly consistently connected systems. To achieve this, however, we need the following modification of the “core principle” of informational redundancy. Suppose that we have two systems, one with connections $\left(R_{1},R_{2},\ldots ,R_{k}\right)$ and the other with connections $\left(R_{0},R_{2},\ldots ,R_{k}\right)$, such that we have both

(22) $$R_{0}=f\left(R_{1},R_{2},\ldots ,R_{k}\right)$$

and

(23) $$R_{1}=g\left(R_{0},R_{2},\ldots ,R_{k}\right).$$

In other words, given the connections $R_{2},\ldots ,R_{k}$, the connections $R_{0}$ and $R_{1}$ are *informationally interchangeable*. Then, we suggest, anyone who considers the principle of informational redundancy intuitively plausible, should also accept the following principle: if a system with $\left(R_{1},R_{2},\ldots ,R_{k}\right)$ is noncontextual, then the system with $\left(R_{0},R_{2},\ldots ,R_{k}\right)$ should be noncontextual too. With the alleged principle thus modified, we can present our counter-example to it by the chain of transformations in which the systems considered at each step are strongly consistently connected:(24)
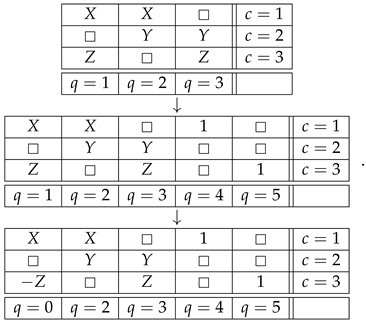
The final transition here is justified by observing that we have both

(25) $$R_{0}=\left\{\begin{array}{ccc} R_{1} & \mathrm{if} & R_{4}=1\\ -R_{1} & \mathrm{if} & R_{5}=1\\ □ & \mathrm{if} & \mathrm{otherwise} \end{array}\right\}=g\left(R_{1},R_{4},R_{5}\right),$$

and

(26) $$R_{1}=\left\{\begin{array}{ccc} R_{0} & \mathrm{if} & R_{4}=1\\ -R_{0} & \mathrm{if} & R_{5}=1\\ □ & \mathrm{if} & \mathrm{otherwise} \end{array}\right\}=h\left(R_{0},R_{4},R_{5}\right).$$

## 8. A Note on Indicator Connections

Many similar examples can be readily constructed. Moreover, for a broad class of systems like those considered in our example one can formulate a general algorithm by which one can introduce new connections using the old connections and some auxiliary ones. The latter prominently include the “indicator connections” each consisting of a single random variable. In our example, we conveniently chose indicator variables as those equal to 1 with probability 1. However, they can be replaced with arbitrary random variables, not necessarily deterministic ones. Consider, for example,
(27)
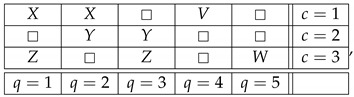

where *V* and *W* are ±1-variables with $p\left[V=1\right]=p\left[W=1\right]=1/2$, chosen so that the system remains noncontextual. For instance, one can introduce *V* as stochastically independent of *X*, and *W* as stochastically independent of *Z*. Alternatively, one could put V=X and W=Z. It is easy to check that in either case the system is noncontextual. Then one can define the new connection $R_{0}$ in the following way:

(28) $$R_{0}=\left\{\begin{array}{ccc} R_{1} & \mathrm{if} & R_{4}=1\;\mathrm{or}\;R_{4}=-1\\ -R_{1} & \mathrm{if} & R_{5}=1\;\mathrm{or}\;R_{5}=-1\\ □ & \mathrm{if} & \mathrm{otherwise} \end{array}\right\}=f\left(R_{1},R_{4},R_{5}\right).$$

The reason this works is that if content *q* is not measured in some context *c*, then the answer to the question

“does the outcome of measuring *q* in *c* equal either 1 or −1?”

must be “no”. Moreover, one can replace “equal to either 1 or −1” with any numerical relation that is always true about a ±1-variable, such as $R_{4}<2$ or $R_{4}\in R$.

## 9. Consistification

If one’s goal is to come up with a statement that would contradict CbD but spare its special case, confined to consistently connected systems, the only way to do this is to somehow prohibit, in formulations of functions, access to information about contexts. One simple way to do this is to only allow functions $R_{0}=f\left(R_{1},\ldots R_{k}\right)$ such that

(29) $$R_{0}=□\;\mathrm{if}\;R_{i}=□\;\mathrm{for}\;\mathrm{some}\;i=1,\ldots ,k.$$

With this constraint, if the old system is consistently connected and noncontextual, so will be the new system, with $R_{0}$ added. What happens here is that the combination of (29) and consistent connectedness amounts to denying contexts any role in determining the distributions of the random variables and functions thereof. Such a constraint, of course, is antithetical to any theory that allows inconsistently connected systems—because, by definition, in such systems measurement outcomes are determined by both contents and contexts. Also, if the claim is that the principle of informational redundancy is intuitively plausible, it is difficult to see how one would justify prohibition of the functions considered in the previous sections of this paper.

Whatever the justification proposed, however, there is an argument against this or any other attempt to dismiss CbD while preserving the theory of consistently connected systems. Consider the following systems:(30)
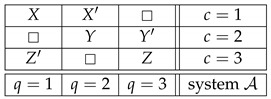

and
(31)
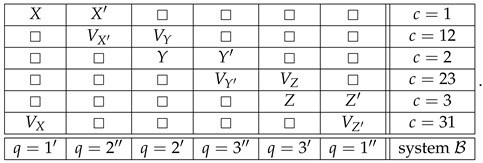
System A is a realization of (1), but this time $X,X^{\prime },Y,Y^{\prime },Z,Z^{\prime }$ are arbitrary dichotomic variables. That is, system A is generally inconsistently connected. System B is a *consistification* of system A, the notion elaborated in Ref. [7], where one can find a general algorithm for consistifying arbitrary systems. (This construction was first described in Ref. [9], except for using maximal rather than multimaximal couplings. This makes no difference for cyclic systems.)

In system B, the bunches of the variables in contexts c=1,2,3 are the same as in system A, although their contents are redefined to make these bunches non-overlapping. There are also new contexts inserted between the old ones, and filled with new random variables. Each variable $V_{T}$ has the same distribution as variable *T* ($=X,X^{\prime },Y,\ldots$), and when in the same context, $V_{T}$ and $V_{U}$ are jointly distributed so that $p\left[V_{T}=V_{U}\right]$ has the maximal possible value (given their individual distributions). Clearly, system B is consistently connected, in fact strongly so.

Now, it can be shown that systems A and B are *contextually equivalent*, in the following sense [7]. *First*, A is contextual if and only if so is B. *Second*, if A is consistently connected, then A and B have the same value of *contextual fraction*, as defined in Ref. [10]. *Third*, whether A is or is not consistently connected, A and B have the same value of any of the CbD measures of contextuality defined in Ref. [2]. (Of these measures, ${\mathrm{CNT}}_{0}$ in A equals ${\mathrm{CNT}}_{2}$ in B because they are computed by the same linear programming algorithm. Since both systems are cyclic (B being cyclic of rank 6), ${\mathrm{CNT}}_{0}={\mathrm{CNT}}_{1}={\mathrm{CNT}}_{2}$ for either of them [2].)

The point of this demonstration is that all systems in CbD can always be redefined in terms of their consistifications, i.e., using strongly consistently connected systems only. For instance, an empirical situation that could be described by system A above can instead be required to be described by system B. If one did this systematically, the resulting theory (let us call it CbD*) would become more cumbersome but would not change in any significant aspect. It is difficult to see how one can find problems with CbD while accepting CbD* if the two formulations are essentially equivalent.

## 10. Concluding Remarks

Rather than continue to elaborate and multiply examples, let us state the main conclusion of this paper, for which a single example is all one needs. In a theory of contextuality based on multimaximally connected couplings, adding an informationally redundant connection or exchanging it for an informationally equivalent one may change a noncontextual system into a contextual one. This is also true for theories confined to consistently connected systems, and even strongly consistently connected systems. Therefore, this fact is inherent to the very notion of contextuality.

The discussion of the connections and their functions in this paper has an unexpected benefit: a better understanding of the role of non-measurements in contextuality analysis. We now know that non-measurements in systems of random variables should be treated as elements of these systems on a par with the measurements represented by random variables. The information provided by a non-measurement, that a given content is not measured in a given context, is accessible, and it can be utilized, in particular, in defining functions mapping connections into connections. In CbD, non-measurements can be replaced with deterministic values, as shown in (2). They can also be viewed as random variables that are equal to the special value □ with probability 1 (see the parenthetical remark at the end of Section 2). For instance, our system (1) is represented by
(32)
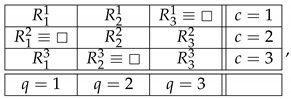

in which every connection contains two distinct distributions. In this sense, every system of interest is inconsistently connected, even if strongly consistently connected in the traditional sense. A strongly consistently connected system without non-measurements is necessarily trivial, with all contexts containing one and the same joint distribution.

Moreover, the inclusion of non-measurements as legitimate values of random variables allows one to extend the notion of a system of random variables to include situations in which a content within a context may sometimes be measured and sometimes not. Consider, for instance, an experiment in which the dichotomous variables $R_{1}^{1}$ and $R_{2}^{1}$ in context c=1 are jointly assigned values (±1) only if the detectors of the corresponding properties “click” within a short temporal window. In a real experiment, it occasionally happens that the two clicks are separated by a larger interval, or one of the two detectors does not click at all. Such experimental results are necessarily excluded from analysis. With the new point of view, however, if a detector does not click or clicks too late, we simply set the corresponding variable equal to □. This means that $R_{1}^{1}$ and $R_{2}^{1}$ (and, by analogy, all other variables in the system) are now trichotomous, with the possible values $\left\{1,-1,□\right\}$ occurring, generally, with non-zero probabilities. This might potentially lead to more comprehensive contextuality analysis, more veridically combining quantum properties with those of the measuring procedures.
